# Tissue-Specific Mercury Bioaccumulation and Probabilistic Human Health Risk in Freshwater Fish from the Arda River Reservoir Cascade (Bulgaria)

**DOI:** 10.3390/toxics14040291

**Published:** 2026-03-28

**Authors:** Violina R. Angelova, Ljudmila N. Nikolova, Stanimir G. Bonev, Georgi K. Georgiev

**Affiliations:** 1 Department of Chemistry, Agricultural University—Plovdiv, 4000 Plovdiv, Bulgaria; 2 Department of Animal Science, Agricultural University—Plovdiv, 4000 Plovdiv, Bulgaria

**Keywords:** mercury, freshwater fish, bioaccumulation, trophic magnification, cascade reservoirs, human health, risk assessment, Monte Carlo simulation, human health risk

## Abstract

Mercury (Hg) bioaccumulation in freshwater fish represents a major pathway of human exposure, particularly in cascade reservoir systems where hydrological retention and legacy contamination can enhance methylmercury (MeHg) formation and trophic transfer. This study quantified total mercury (THg) concentrations in seven tissues of seven fish species from the Arda River cascade (Bulgaria). Multi-tissue measurements were integrated with morphometric predictors, multivariate statistical analyses, and combined deterministic and probabilistic human-health risk assessments. Muscle and liver contained the highest THg concentrations, whereas gills and gonads exhibited the lowest levels. Predatory species and larger individuals accumulated significantly more Hg, reflecting trophic magnification and size-dependent exposure. A longitudinal gradient across the cascade reservoirs suggests hydrological retention effects influencing mercury distribution. Species- and tissue-specific size–Hg relationships further indicate heterogeneous bioaccumulation dynamics among taxa. Risk assessment indicated acceptable exposure for adults and pregnant women at average consumption (140 g·week^−1^), but elevated exposure for children consuming high-Hg predators. Monte Carlo simulations (*N* = 30,000) revealed upper-tail risks, while Safe Weekly Intake thresholds provided species-specific consumption limits. These findings highlight the value of integrating multi-tissue monitoring with probabilistic risk modelling to support evidence-based fish-consumption advisories in contaminated freshwater systems.

## 1. Introduction

Mercury (Hg) is among the most significant global priority pollutants due to its persistence, volatility, and ability to undergo intercontinental atmospheric transport. Elemental mercury (Hg^0^) has an atmospheric lifetime of up to one year, allowing transboundary transport and deposition in regions distant from primary emission sources [1,2]. Following oxidation to divalent mercury (Hg^2+^), the element becomes incorporated into hydrological and biogeochemical cycles through wet and dry deposition, entering aquatic ecosystems where it undergoes dynamic exchanges among the water column, sediments, and biota [3,4].

A key process determining the toxicity and ecological significance of Hg is microbial methylation. In anaerobic sediments, Hg^2+^ is transformed into methylmercury (MeHg) by sulfate- and iron-reducing bacteria [5,6]. MeHg is a highly toxic, lipophilic, and biologically stable form that readily crosses the blood–brain and placental barriers [7,8]. As a result, MeHg dominates aquatic food webs and represents the primary form of mercury accumulated in fish.

The intensity of methylation is controlled by environmental factors including organic matter availability, sulfide chemistry, redox potential, temperature, and water-column stratification [9,10,11]. Recent studies suggest that internal biogeochemical processes can maintain elevated MeHg concentrations even under declining atmospheric Hg emissions, particularly in stratified lakes and reservoir systems [12,13,14]. This finding challenges the assumption of a direct relationship between emission reductions and decreasing exposure in aquatic organisms.

The global concern regarding mercury pollution led to the adoption of the Minamata Convention on Mercury [2]. Within the European Union, maximum permissible concentrations of Hg in fish are regulated by Commission Regulation (EU) 2023/915 [15]. However, regulatory thresholds do not always reflect cumulative exposure resulting from high consumption frequencies or the increased vulnerability of sensitive population groups such as children and pregnant women [16,17].

Fish are widely recognised as reliable bioindicators of metal contamination in freshwater ecosystems because they integrate exposure across the water column, sediments, and trophic transfer pathways [18,19]. In reservoir systems characterized by slow water exchange, the accumulation of organic matter and sediment remobilisation can enhance Hg retention and transformation processes [9]. Mercury concentrations in fish tissues frequently exceed those in surrounding waters, with bioaccumulation largely determined by trophic level, age, size, and feeding strategy [20,21,22].

Trophic position is a key predictor of Hg concentrations due to biomagnification within aquatic food webs [19]. Predatory species with longer lifespans typically accumulate higher mercury concentrations as a result of cumulative dietary exposure [21]. In parallel, body size and age strongly influence bioaccumulation patterns, although species-specific variability may arise from differences in growth rates, metabolism, and ecological behaviour [23,24,25].

Organ-specific distribution of Hg provides important insight into accumulation mechanisms. Muscle tissue represents the primary storage compartment and the most relevant indicator of dietary exposure in humans [26,27], whereas the liver and kidneys reflect physiological processes related to metabolism and detoxification [28,29]. Recent multi-tissue assessments of fish bioindicators further highlight the value of tissue-level profiling for understanding mercury dynamics and associated ecological and human-health risks [30].

The Arda River dam cascade (Kardzhali, Studen Kladenets, and Ivaylovgrad) represents a hydrologically connected reservoir system influenced by a historical Pb–Zn mining legacy, where sediments may act as secondary sources of Hg. Despite the environmental relevance of such systems, integrated analyses simultaneously addressing interspecific variability, organ-specific distribution, and quantitative risk assessment in cascade reservoirs remain scarce.

Relative to previous regional investigations focused on a single reservoir [31], the present study adopts a cascade-wide perspective combining multi-tissue mercury profiling with an integrated deterministic–probabilistic risk framework (THQ, SWI, and Monte Carlo simulation).

Based on these knowledge gaps, the study tests the following hypotheses:

**H1**— *predatory species exhibit higher Hg concentrations*;

**H2**— *morphometric metrics (TL and TW) are significant predictors with organ-specific variability*;

**H3**— *organ distribution patterns are statistically significant and physiologically grounded*;

**H4**— *spatial differences are detectable but secondary to biological determinants*.

The objectives of this study are to: (i) quantify organ-specific Hg concentrations in freshwater fish from the Arda cascade; (ii) evaluate spatial variability among reservoirs; (iii) analyse morphometric determinants of Hg accumulation; and (iv) perform integrated deterministic and probabilistic human-health risk assessments. The novelty of this work lies in combining multi-tissue analysis, trophic–morphometric structuring, and probabilistic exposure modelling within a cascade reservoir framework—an approach still rarely applied in freshwater ecosystems of Southeastern Europe and one that contributes to a more mechanistic understanding of Hg dynamics in hydrologically complex systems.

## 2. Materials and Methods

### 2.1. Study Area

The study was conducted in the three major reservoirs of the Arda River cascade in southern Bulgaria: Kardzhali (upper reservoir), Studen Kladenets (middle reservoir), and Ivaylovgrad (downstream reservoir). Together, these reservoirs form a hydrologically connected longitudinal system along the Arda River continuum. Cascade reservoir systems often develop spatial gradients in pollutant transport, sedimentation, and transformation processes, with upstream basins frequently acting as biogeochemical filters that retain particulate matter and associated contaminants.

The study region has a long history of Pb–Zn mining and metallurgical activities, which have resulted in legacy contamination of surrounding soils and riverine sediments. These historical inputs may contribute to secondary mercury mobilization within the reservoir system. Pb–Zn ore processing typically generates sulfide-rich tailings that enhance mercury binding in sediments, while subsequent geochemical changes can promote remobilization under certain redox conditions. In addition, historical smelting activities are known to release volatile mercury to the atmosphere, providing an additional pathway for regional Hg deposition.

**Figure 1:**
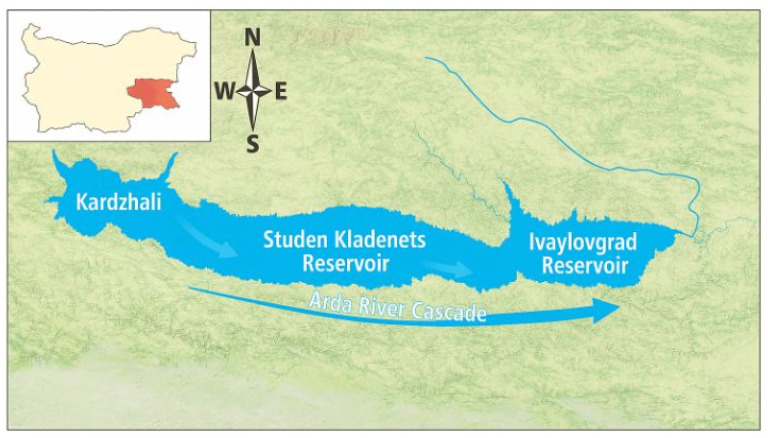
Map of the study area (Arda River reservoir cascade). Schematic layout of the Kardzhali (upstream), Studen Kladenets (midstream) and Ivaylovgrad (downstream) reservoirs.

The geographic locations of the reservoirs and the overall configuration of the cascade system are presented in Figure 1.

### 2.2. Fish Sampling and Species Composition

Fish samples were collected between 2022 and 2024 with the assistance of licensed fishermen and in compliance with national animal welfare regulations. A total of 49 specimens representing seven freshwater fish species were obtained using hook-and-line fishing methods. Captured individuals were transported in refrigerated containers and processed within 24 h of capture.

Each specimen was identified to species level using standard ichthyological identification keys [32,33]. Morphometric parameters, including total length and total weight, were recorded prior to tissue sampling. The distribution of species across reservoirs and trophic categories is presented in Table 1, while detailed sampling counts by species and reservoir (species × reservoir, n) are provided in Table S1 to ensure full reproducibility of specimen allocation.

Sampling was conducted between late spring and early autumn under comparable hydrological conditions. Although seasonal variability in mercury methylation and retention processes may influence bioaccumulation dynamics, potential seasonal effects are addressed in the Section 4.9.2.

The selected species represent key trophic guilds within the reservoir ecosystem and include both predatory taxa (e.g., perch and European catfish) and omnivorous species (e.g., Prussian carp, roach, and Orpheus dace). This design enables the evaluation of trophic influences on mercury accumulation while reflecting fish species commonly consumed by local populations.

### 2.3. Morphometrics Measurements

Total length (TL, cm) and body mass (TW, g) were measured for each specimen with an accuracy of ±0.1 cm and ±0.01 g. To analyse size-related effects on bioaccumulation, *Carassius gibelio* was divided into two size classes based on the median TW.

### 2.4. Sample Preparation and Mercury Analysis

After transport, fish were rinsed with distilled water (Milli-Q purification system, Merck Millipore, Burlington, MA, USA), dried, and dissected. Muscle (dorsal part), liver, kidneys, spleen, gills, bones, and gonads were separated. Tissues were homogenised, and approximately 100 mg of each sample was analysed for total mercury (THg) using a direct mercury analyser MA-3000 (Nippon Instruments Corporation, Kyoto, Japan) according to US EPA Method 7473 [34], based on thermal decomposition, amalgamation, and atomic absorption spectrometry at 253.7 nm.

Quality control included a six-point calibration (R^2^ ≥ 0.999), triplicate measurements, reagent blanks, and analysis of certified reference materials BCR-463 (Joint Research Centre, European Commission, Bruxelles, Belgium). The limit of detection (LOD) was 0.05 µg·kg^−1^ (0.00005 mg·kg^−1^).

### 2.5. Statistics Analysis

All statistical analyses were performed in R v4.3.1 (R Core Team, 2023; R Foundation for Statistical Computing, Vienna, Austria) using RStudio 2023.06 (RStudio, PBC, Boston, MA, USA). Data processing, visualisation, and statistical modelling were conducted using the packages stats, dplyr, ggplot2, EnvStats, corrplot, PerformanceAnalytics, and fitdistrplus.

Data normality was assessed using the Shapiro–Wilk test, and log_10_ transformation was applied where necessary to improve distributional assumptions. Differences in mercury concentrations among species, organs, and reservoirs were evaluated using one-way analysis of variance (ANOVA) followed by Tukey’s HSD post hoc test. Homogeneity of variances was verified prior to ANOVA.

Associations between morphometric variables and mercury concentrations were examined using Pearson correlation for normally distributed variables. Spearman rank correlations were additionally calculated to assess monotonic relationships between mercury concentrations and biological predictors, including total length (TL), total weight (TW), and trophic level. Correlation matrices were visualised as heatmaps to facilitate interpretation of multivariate patterns related to body size and trophic structure.

Intraspecific variability was quantified using the coefficient of variation (CV, %). Statistical significance was defined at *p* < 0.05. 

Size–Hg relationships were further modelled using ordinary least squares (OLS) regression, with muscle THg as the response variable and TL and TW as predictors. Models were fitted by species and, where sample size permitted, by reservoir. Diagnostic evaluation included residual analysis, leverage assessment, and tests of variance homogeneity. Effect sizes (r), significance levels (*p*), and sample sizes (*n*) are reported, with additional diagnostics presented in Figures S6 and S7.

### 2.6. Human Health Risk Assessment

Human health risk assessment was conducted using both deterministic and probabilistic approaches. The average fish consumption rate for the Bulgarian population (7.3 kg·year^−1^, corresponding to 140 g·week^−1^) [35] was applied as the baseline exposure scenario. For the toxicological reference benchmarks, we used the U.S. EPA oral reference dose for methylmercury (RfD = 0.1 µg·kg^−1^·day^−1^; US EPA, 2001) and the EFSA tolerable weekly intake (TWI = 1.3 µg·kg^−1^·week^−1^).

#### 2.6.1. Estimated Daily Intake (EDI)

EDI (mg·kg^−1^·day^−1^) was calculated as:

$$EDI\;=\frac{C\times IR}{BW}$$

where C is the concentration of THg in muscle (mg·kg^−1^ ww), IR is the daily intake (g·day^−1^), and BW is body weight (70 kg for adults, 60 kg for pregnant women, and 15 kg for children).

#### 2.6.2. Target Hazard Quotient (THQ)

$$THQ=\frac{EDI}{RfD}$$

where RfD = 0.1 µg·kg^−1^·day^−1^ (=0.0001 mg·kg^−1^·day^−1^). THg was used as a proxy for MeHg, as 80–95% of Hg in muscle tissue is methylmercury [16]. THQ values < 1 are interpreted as indicating no significant non-carcinogenic risk. While muscle Hg is predominantly MeHg (often 80–95%), occasional deviations below 60% have been reported; thus, we acknowledge residual uncertainty when using THg as a proxy for MeHg.

#### 2.6.3. Hazard Index (HI)

HI represents the cumulative non-carcinogenic risk and was calculated as the sum of individual THQs when more than one fish species is consumed. The index was estimated using both deterministic calculations and probabilistic modelling via Monte Carlo simulation (30,000 iterations). In the probabilistic approach, fish ingestion rate (IR) was sampled from a triangular distribution, IR~Tri(50, 140, 350) g·week^−1^; mercury concentration (C) was resampled by non-parametric bootstrap; and body weight (BW) was treated as a fixed parameter for each consumer group. The triangular distribution was selected because it is appropriate under data-limited conditions and allows incorporation of minimum, most-likely, and maximum ingestion scenarios.

#### 2.6.4. Tolerable Weekly Intake (TWI) and Safe Weekly Intake (SWI)

EFSA [16] defines a TWI for MeHg of 1.3 µg·kg^−1^·week^−1^. The Safe Weekly Intake (SWI) was calculated as:

$$SWI\;=\frac{TWI\;\;\times \;\;BW}{C}\;\;\times \;\;1000$$

where TWI = 1.3 µg·kg^−1^·week^−1^ (i.e., 0.0013 mg·kg^−1^·week^−1^), BW is body weight (70 kg for adults, 60 kg for pregnant women, and 15 kg for children), and C is the measured THg concentration in muscle (mg·kg^−1^ ww).

SWI translates toxicological thresholds into practical consumption limits (g·week^−1^) and supports transparent risk communication, particularly for vulnerable groups. In this study, SWI was calculated using both species-wise median and P95 muscle THg concentrations. A traffic-light baseline was set at 7.3 kg·year^−1^ (140 g·week^−1^) [35], with categories defined as: Red < 140 g·week^−1^, Yellow = 140–299 g·week^−1^, and Green ≥ 300 g·week^−1^. Species-wise SWI for children is summarised in Figure S1A,B.

#### 2.6.5. Monte Carlo Simulation

A Monte Carlo simulation with 30,000 iterations was used to model exposure variability. Muscle THg concentrations (C, mg·kg^−1^ ww) were sampled using species-specific empirical bootstrap resampling. Fish ingestion rate (IR) was drawn from a triangular distribution, IR~Tri(50, 140, 350) g·week^−1^ (converted to daily intake for THQ), while body weight (BW) was fixed at 70, 60, and 15 kg for Adults, Pregnant women, and Children, respectively. The triangular distribution was selected because it is suitable under data-limited conditions and allows the incorporation of minimum, most-likely, and maximum ingestion scenarios.

For each iteration, THQ was computed and summarised using the median (P50), upper percentile (P95), and exceedance probability P(THQ > 1). Species- and group-specific results are presented in S6, with PDF/CDF visualisations in Figures S2 and S3. 

In the probabilistic framework, IR was sampled from IR~Tri(50, 140, 350) g·week^−1^ to reflect data-limited intake with expert-informed minimum, most-likely, and upper-bound scenarios; C was resampled via species-specific empirical bootstrap to preserve empirical skewness and tails without imposing a parametric form. C and IR were sampled independently as a transparent baseline; potential dependence (e.g., species-targeted consumption) is addressed in the Limitations.

### 2.7. Data Presentation

Data are presented as mean ± SD for normally distributed variables and median (min–max) for non-normal distributions. Visualisations include boxplots, scatterplots, correlation matrices, TL/TW heatmaps, and forest plots.

## 3. Results

Mercury concentrations differed significantly among species, tissues, and reservoirs, revealing distinct trophic, morphometric, and spatial patterns across the Arda River cascade. Results are organised around species-specific mercury variability, size relationships, and multivariate correlation patterns.

### 3.1. General Levels and Variability of Mercury

**Figure 2:**
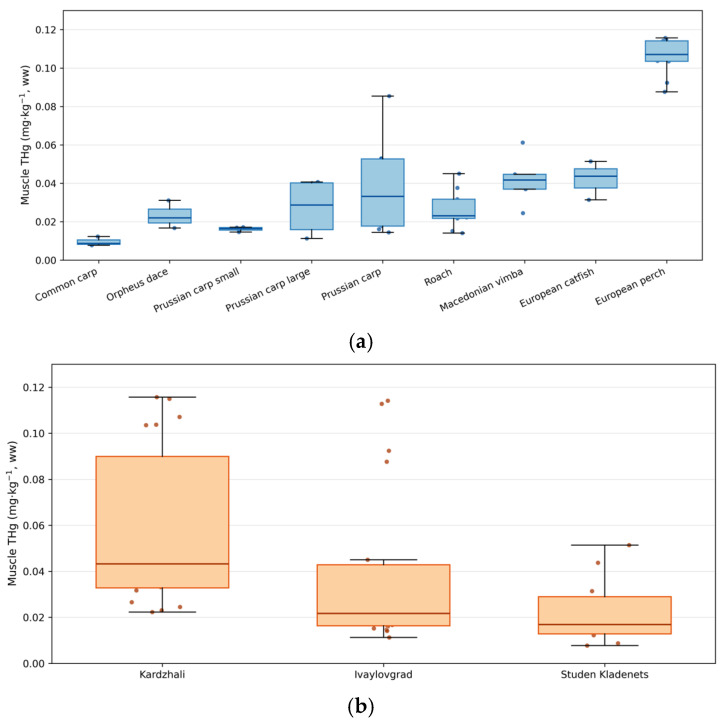
Muscle THg (mg·kg^−1^, ww) distributions across species and reservoirs. (**a**) Species-specific muscle THg (box/strip, points = individuals); (**b**) Spatial gradient of muscle THg across reservoirs (Kardzhali → Ivaylovgrad → Studen Kladenets).

A total of 49 fish specimens representing seven species were analysed (Table 1). Mercury concentrations (THg, mg·kg^−1^ wet weight) showed pronounced species- and tissue-specific variability (Table 2). The highest concentrations were observed in muscle and liver tissues, whereas gills and gonads exhibited the lowest levels. The distribution of muscle mercury concentrations is presented in Figure 2, while organ–species patterns are summarised in Table S2 and Figure S4A,B.

**Figure 3:**
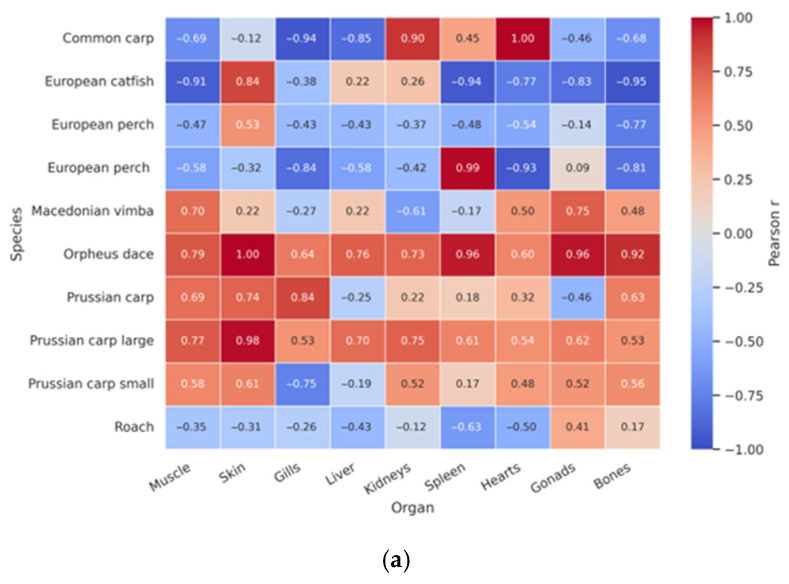

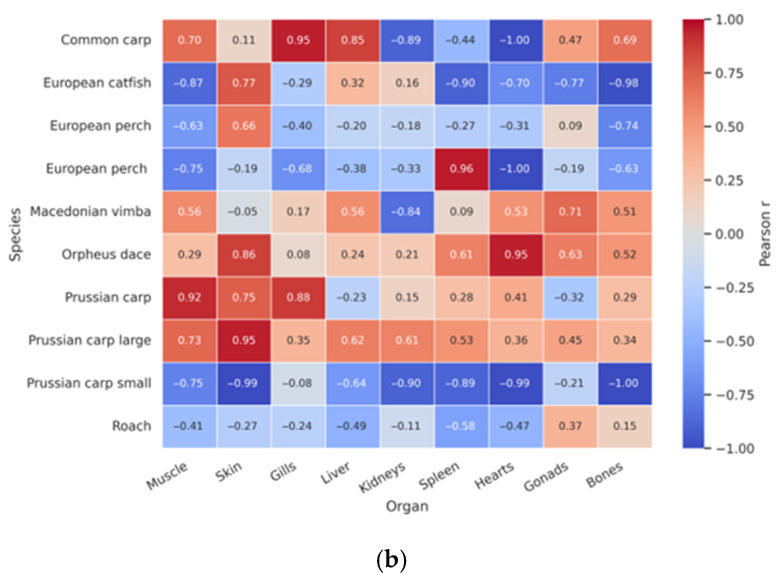
Correlation heatmaps between morphometric predictors (TL, TW) and tissue THg across species: (**a**) TL-Hg correlation heatmap (species × organs); (**b**) TW–Hg correlation heatmap (species × organs).

**Figure 4:**
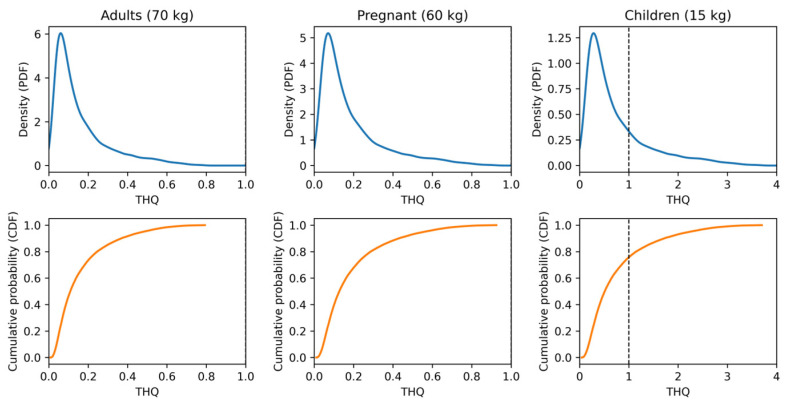
Monte Carlo THQ distributions (*N* = 30,000): consolidated panel with the THQ threshold (THQ = 1). Top: PDF; Bottom: CDF. Dashed line indicates THQ = 1. IR~Tri(50, 140, 350) g·week^−1^; RfD = 0.1 µg·kg^−1^·day^−1^. (**Top**) PDF; (**Bottom**) CDF; dashed line at THQ = 1. IR~Tri(50, 140, 350) g·week^−1^; RfD = 0.1 µg·kg^−1^·day^−1^.

A clear longitudinal gradient across the reservoir cascade was observed (Kardzhali > Ivaylovgrad > Studen Kladenets), as illustrated in Figure 3. Pearson TL–Hg relationships across species and tissues are presented in Figure 4, with extended correlation matrices provided in Figures S4 and S5. Mean muscle THg concentrations were 0.058, 0.039, and 0.014 mg·kg^−1^ ww for Kardzhali, Ivaylovgrad, and Studen Kladenets, respectively. Extended visualisations of tissue × reservoir distributions are provided in Figure S5A,B.

Muscle mercury concentrations differed significantly among species (one-way ANOVA, *p* < 0.05). The highest median concentrations (mg·kg^−1^ ww) were recorded in perch (0.107) and European catfish (0.044), whereas Prussian carp (0.018) and common carp (0.009) exhibited the lowest values (Table 2). Predatory species generally showed higher mercury concentrations across tissues, consistent with trophic biomagnification patterns.

Correlation heatmaps further differentiated storage tissues (muscle, liver) from metabolically active or barrier tissues (gills, gonads). Diagnostics of TL–Hg relationships are presented in Figure S7, while species-specific TW–Hg contrasts are shown in Figure S6. Probabilistic exposure summaries are reported in Table 4B and Table S6, with additional spatial context provided in Figure S5A,B.

### 3.2. Species-Specific Concentrations

Organ–species profiles (mean THg, mg kg^−1^ wet weight) indicated a dominant influence of trophic position and storage tissues (muscle and liver). Principal component analysis (PCA) and hierarchical clustering further supported these patterns. Muscle mercury concentrations differed significantly among species (one-way ANOVA, *p* < 0.05; Table 2).

Predatory species, including *Perca fluviatilis* and *Silurus glanis*, consistently exhibited higher THg levels across tissues compared to omnivorous species (*Carassius gibelio, Rutilus rutilus, Squalius orpheus*). A clear spatial gradient was observed along the cascade, with higher concentrations in upstream reservoirs (Figure 3).

Size-structured relationships between total length (TL), total weight (TW), and THg concentrations are summarised in Figure 3. Supporting multivariate analyses, including tissue × species PCA and tissue × reservoir comparisons, are provided in Figures S4A,B and S5A,B.

### 3.3. Spatial Differences Between Dams

Significant differences in mercury concentrations were observed among the three reservoirs (one-way ANOVA, *p* < 0.05; Table 2). Muscle THg exhibited a longitudinal gradient along the cascade, with mean concentrations of 0.058, 0.039, and 0.014 mg·kg^−1^ ww in Kardzhali, Ivaylovgrad, and Studen Kladenets, respectively. Tissue × reservoir patterns and contrasts are summarised in Figure S5A,B.

The spatial effect was most pronounced in predatory species, suggesting a trophic–spatial interaction. Species-specific, size-dependent relationships between total weight (TW), total length (TL), and THg across reservoirs are shown in Figure S6A,B, while multivariate tissue-level structure across reservoirs is visualised in hierarchical cluster maps (Figure S4A,B). Comprehensive ANOVA outputs for the tissue × reservoir factor are provided in Table S4.

These spatial differences likely reflect hydrological and biogeochemical features typical of cascade systems: hydrological retention fostering accumulation of fine sediments and organic matter in depositional zones (favouring microbial methylmercury formation), longer water residence times enhancing Hg transformations and bioavailability, and seasonal stratification with low-oxygen bottom waters stimulating sedimentary Hg methylation—jointly contributing to reservoir-specific variability in bioavailable mercury.

### 3.4. Organ-Specific Distribution

Organ-level mercury contrasts reflected underlying physiological drivers of accumulation. Muscle and liver consistently exhibited the highest THg concentrations, whereas gills and gonads showed the lowest levels across species. Heatmaps visualising size–tissue relationships are presented in Figure 3 and Figure S4A,B.

Species-specific correlations between total length (TL) and tissue THg further highlighted organ-dependent patterns. In *Carassius gibelio*, TL was positively associated with muscle THg (r = 0.624, *p* = 0.023), whereas *Perca fluviatilis* exhibited negative TL–Hg correlations in certain tissues, such as bone (r = −0.760, *p* = 0.018). Numerical summaries of organ-specific concentrations and correlation estimates are provided in Table 3 and Tables S3 and S4, with a consolidated TL–Hg overview shown in Figure 3. Additional correlation metrics supporting organ-level contrasts across species are reported in Table S3.

### 3.5. Spatially Modified Size Dependencies

At the whole-dataset level, TL–Hg correlations are summarised in Figure 3, with reservoir-specific scatterplots shown in Figure S6A,B. Patterns varied spatially: Studen Kladenets exhibited predominantly positive relationships (e.g., liver r = 0.751; spleen r = 0.710; gonads r = 0.699), whereas Kardzhali showed negative trends in barrier tissues (e.g., gills r = −0.494; gonads r = −0.552). The trophic hierarchy remained consistent (predators > omnivores > benthic species). A compact overview across species × reservoirs is provided in Figure S7.

Within Ivaylovgrad, Prussian carp analysed in two size classes (“small” vs. “large”) exhibited higher muscle THg in the large class (median 0.0288 vs. 0.0164 mg·kg^−1^ ww; 1.8-fold; Mann–Whitney U = 4.0, *p* = 0.343). Monte Carlo THQ indicated P(THQ > 1) = 6.5% for children in the large class (P50 = 0.40, P95 = 1.04), whereas other groups remained <1. Correspondingly, SWI values were higher for smaller fish. Although underpowered (*n* = 4 vs. 4), these results support size-selective advisories favouring smaller individuals from Ivaylovgrad. Species-specific TL–Hg contrasts for Prussian carp are shown in Figure S7A, while split scatterplots for *Perca fluviatilis*, *Carassius gibelio*, and *Rutilus rutilus* are shown in Figure S7B.

Split-by-reservoir scatterplots (Figures S6A,B and S7A,B) further confirmed that TL/TW–Hg associations were spatially modified. At Kardzhali, predator signals were stronger, whereas at Studen Kladenets, positive size effects were also evident in non-predatory taxa. Analyses were restricted to panels with *n* ≥ 3.

### 3.6. Risk Assessment (THQ, SWI, Monte Carlo)

Deterministic THQ and SWI estimates are presented in Table 4, while probabilistic outputs are summarised in Table 4 and Table S6. At the national average fish intake of 140 g week^−1^, THQ remained <1 for adults and pregnant women across most species, whereas predatory taxa approached or exceeded unity in children. SWI values reflected trophic-level differences, with predators exhibiting the lowest safe-intake thresholds. Based on the EFSA TWI of 1.3 µg kg^−1^ week^−1^, SWI ranged from 860–9502 g week^−1^ in adults, 737–8145 g week^−1^ in pregnant women, and 184–2036 g week^−1^ in children. Relative to the national intake of 140 g week^−1^, low-Hg species remained within safe limits for adults, whereas predatory fish may exceed safety thresholds in children. Visual SWI guidance for children is provided in Figure S1A,B.

Monte Carlo simulations (*N* = 30,000) produced right-skewed THQ distributions with elevated P95 values in predatory species, especially under high-intake scenarios. Consolidated PDF/CDF outputs are shown in Figure 4, with species-wise probability suites in Figure S2, and exceedance probabilities summarised in Table 4 and Table S6. Forest-plot synthesis (Figure S8, Supplement) highlights concentrated exceedance risks P(THQ > 1) in children. In the probabilistic framework, IR was sampled from a triangular distribution Tri(50, 140, 350) g week^−1^, C was resampled by empirical bootstrap, and BW was fixed for each consumer group; the triangular distribution was selected because it is suitable under data-limited conditions and captures minimum, most-likely, and maximum intake scenarios.

## 4. Discussion

The present study integrates multi-tissue mercury measurements, morphometric predictors, and probabilistic exposure modelling to elucidate the ecological and human-health implications of Hg accumulation across the Arda River cascade system. The findings are synthesised by mapping the results onto hypotheses H1–H4, followed by a discussion of their implications for risk assessment and environmental management. In contrast to prior work focused on a single reservoir and multi-metal screening in the region [31], the present study resolves cascade-wide patterns with multi-tissue profiling and integrated probabilistic risk assessment.

### 4.1. Spatial Gradient and Cascade Reservoir Systems (H1)

THg concentrations in muscle tissue along the Arda River cascade confirm a pronounced spatial gradient (Kardzhali > Ivaylovgrad > Studen Kladenets), best resolved in muscle—the tissue providing the most stable long-term record of dietary MeHg exposure (Figure 3; Table 2). Although THg was quantified in multiple organs, organ-specific datasets were not sufficiently balanced across reservoirs to resolve an equally robust spatial pattern.

This gradient aligns with cascade-reservoir theory, which posits that upper reservoirs act as biogeochemical “filters,” retaining sediment-bound loads and promoting internal transformations [36,37]. Elevated values in Kardzhali are consistent with legacy metal loading and remobilisation from mining and metallurgical activities [38,39,40], as well as with environmental conditions that enhance methylation—prolonged stratification, elevated organic matter, and strong redox gradients [9,10,11,13].

Despite the statistical significance of the spatial effect, interspecific and organ variability exceeded inter-reservoir variability, highlighting the dominant role of biological determinants (trophic position, morphometry, tissue physiology) [20,41]. Under future climate scenarios, stronger and longer stratification combined with warming is likely to modify Hg methylation dynamics and bioavailability [12,42].

### 4.2. Trophic Biomagnification and Species Contrasts (H2)

The observed hierarchy Predators > Omnivores in muscle (Figure 3; Table 2) is characteristic of freshwater food webs and reflects cumulative trophic transfer of MeHg to higher trophic levels and older individuals [19,21,43]. Higher medians in *Perca fluviatilis* and *Silurus glanis* relative to carp and roach are consistent with isotopically and ecologically grounded models of biomagnification [44,45]. The multidimensional clustering of muscle and liver as high-accumulation depot compartments further supports a dominant dietary uptake pathway [46,47].

### 4.3. Organ-Specific Toxicokinetics and Internal Dynamics (H3)

Organ-level differences were pronounced (ANOVA, *p* < 0.05), with muscle and liver showing the highest concentrations and gills and gonads the lowest (Table 2; Figure S5A,B). The predominance of MeHg in muscle (80–95%) explains its stable accumulation [8,16,26]. Liver variability likely reflects active demethylation and binding to sulfhydryl-containing biomolecules [27,28,48]. Kidneys preferentially accumulate inorganic Hg [49], whereas low concentrations in gonads and gills are consistent with barrier function and a predominantly dietary exposure pathway [18,29]. The Organ × Species heatmap (Figure S4A,B) illustrates clear compartmentalisation consistent with established toxicokinetic models.

### 4.4. Size-Dependent Bioaccumulation and Growth Dilution (H4)

Size effects were species- and tissue-specific. Positive TL/TW–Hg relationships (e.g., in *Carassius gibelio*) indicate cumulative exposure and possible ontogenetic trophic shifts, whereas negative relationships (e.g., perch bone) are consistent with growth dilution and/or intra-tissue redistribution [22,23,24,29,50]. Extended correlation matrices (Tables S3 and S4) confirm that TL is a strong predictor in specific species × tissue combinations but not universally—an important caveat for interpretation (Figure S7A,B; Table 3). In particular, Table S3 details the organ-specific TL/TW–Hg associations that underpin these size-structured responses.

### 4.5. Spatially Modified Size Effects

Reservoir-specific models indicate that local conditions modulate morphometric predictors. At Studen Kladenets, positive TL–Hg relationships were observed in metabolic tissues, whereas at Kardzhali, negative trends appeared in barrier tissues (Table S4; Figure S6A,B). These patterns are consistent with the influence of DOC complexation and terrestrial MeHg sources [10,51], as well as the central role of sulfate-reducing bacteria in methylation [6]. Despite local modulation, the trophic hierarchy Predators > Omnivores remained the dominant organising pattern (Figure 3; Table 2). Complementary panel views are provided in Figure S7A,B, which summarise species-focused TL–Hg contrasts across reservoirs.

### 4.6. Implications for Human Health: An Integrated Approach (THQ + SWI + MC)

Deterministic THQ estimates remained <1 for most species in adults and pregnant women at the average intake of 140 g week^−1^, whereas in children, THQ values for predatory species approached or exceeded unity (Table 4, Main; Figure S1A,B, Supplement). SWI values (g week^−1^) translate toxicological thresholds into species- and group-specific consumption limits, with the strictest constraints observed in children. These findings are consistent with the EFSA TWI of 1.3 µg kg^−1^ bw week^−1^ [16] and established WHO/US EPA risk-assessment frameworks [52,53].

Monte Carlo simulations (*N* = 30,000) provide a probabilistic dimension to the assessment, capturing variability through P50/P95 estimates and exceedance probabilities P(THQ > 1). Under extreme intake scenarios, even species with moderate Hg levels may pose elevated risks [54,55,56]. The integration of THQ, SWI, and Monte Carlo outputs yields a regulatory-relevant and communicable exposure profile (Figure 4; Table 4 and Table S6). Species-wise THQ suites are provided in Figure S2, while HI probability density and CDF patterns are presented in Figure S3A,B. Collectively, these visualisations highlight the higher exceedance risks P(THQ > 1) for children consuming predatory species, underscoring the need for species-specific consumption guidance alongside SWI-based thresholds.

In the probabilistic framework, IR was sampled from a triangular distribution Tri(50, 140, 350) g week^−1^, C was resampled by empirical bootstrap, and BW was held constant for each consumer group. The triangular distribution was selected because it is suitable under data-limited conditions and incorporates minimum, most-likely, and upper-bound ingestion scenarios.

### 4.7. Supplement: Mixed Menus (HI)—Supplementary Only

The Hazard Index (HI = ΣTHQi) for mixed weekly menus is presented in the Supplementary Materials, including deterministic and Monte Carlo outputs (Table S7; Figure S2). Diet baskets dominated by predatory species yielded substantially higher HI values, particularly in children. The probabilistic distributions were strongly right-skewed, with elevated P95 and P(HI > 1), highlighting the importance of species composition and consumption frequency in cumulative exposure [16,53].

### 4.8. Ecosystem Modulators and Management Implications

Environmental modulators such as DOC, sulfate chemistry, pH/temperature, and water-column stratification exert strong control over Hg methylation and bioavailability. The observed spatial gradient across the cascade underscores the need for locally calibrated assessments [1,3,6,11]. From a management perspective, SWI-based consumption guidance adjusted for body weight, age, and trophic level is preferable to generic advisories. Traffic-light visualisations combined with probabilistic outputs facilitate risk communication for vulnerable groups and regulatory stakeholders (Figure S1A,B; Table 4A,B, Tables S6 and S7). The concentration of P(THQ > 1) in children consuming high-Hg predators is clearly demonstrated by the integrated Monte Carlo distributions (Figure 4; Table 4B and Table S6), reinforcing the need for species-specific consumption guidance alongside SWI.

### 4.9. Strengths, Limitations, and Future Work

#### 4.9.1. Strengths

This study integrates a multi-tissue and multi-species design, combining organ-specific THg measurements with detailed morphometric data and multi-variate visualisation. The joint use of deterministic (THQ) and probabilistic (Monte Carlo) approaches, alongside SWI-based consumption thresholds, provides a comprehensive risk-assessment framework aligned with established regulatory benchmarks [16,52,53]. This integrated structure strengthens both mechanistic interpretation and management relevance.

#### 4.9.2. Limitations

Several constraints should be acknowledged. First, THg was used as a proxy for MeHg, introducing uncertainty despite the typically high MeHg fraction in muscle (80–95%). Second, the dataset lacks seasonal coverage, limiting assessment of temporal variability. Third, sample sizes for specific species–organ combinations were limited, reducing statistical power. Finally, the ingestion scenarios may not fully capture individual variability in real-world consumption patterns. Small sample sizes in some species (e.g., *n* = 3) may reduce bootstrap stability; BW was treated as fixed (70/60/15 kg), which does not capture full population variability—future work should consider BW distributions.

#### 4.9.3. Future Work

Future research should include direct quantification of MeHg and expanded seasonal and ontogenetic sampling to capture temporal and developmental dynamics. Advanced mixed-effects modelling (e.g., logHg ~ TL + TW + (1|Species) + (1|Location) + (1|Organ)) could better disentangle hierarchical sources of variation. Incorporating locally realistic diet-basket scenarios into HI calculations, combined with population-specific consumption profiles, would further refine risk characterisation [16,52,54].

## 5. Conclusions

The integrated analysis—combining empirical THg measurements across multiple tissues, regression modelling, morphometric–toxicological correlations, and probabilistic Monte Carlo simulations (*N* = 30,000)—demonstrates that fish size (particularly total length), species identity, and organ-specific accumulation jointly determine mercury burdens in cascade-type reservoir systems. Predatory taxa such as perch and European catfish, as well as larger individuals across species, consistently exhibited higher THg concentrations, reflecting trophic magnification and cumulative dietary exposure. The right-tailed THQ distributions indicate non-negligible exceedance risks under high-intake scenarios (e.g., upper-quantile consumers with intakes well above the national average), with children representing the most vulnerable group.

Organ-level patterns confirm muscle as the principal long-term methylmercury depot and the liver as a metabolically active and more variable accumulation site, supporting the value of multi-tissue study designs in ecotoxicological monitoring. Trophic position remains the dominant driver of interspecific differences, while hydrological position along the cascade imposes a secondary but measurable spatial gradient (Kardzhali > Ivaylovgrad > Studen Kladenets). Size-associated effects are detectable but species- and context-dependent rather than universally linear, underscoring the need to incorporate biological traits and local ecological conditions into contaminant-modelling frameworks.

Deterministic exposure metrics suggest generally acceptable non-carcinogenic risk at average national intake levels (140 g week^−1^, corresponding to 7.3 kg year^−1^) [33] for adults and pregnant women. However, probabilistic outputs reveal elevated upper-tail risks for children and for consumers of predatory species, highlighting the limitations of relying solely on point estimates. These patterns reinforce the importance of combining THQ-based evaluation with SWI-derived consumption thresholds, which provide clear, actionable, and population-sensitive guidance on allowable weekly fish intake.

Overall, coupling multi-organ profiling with deterministic and probabilistic risk-assessment approaches provides a robust, decision-ready evidence base for developing species-specific, size-aware, and socially responsive fish-consumption advisories in regulated freshwater systems, particularly those affected by legacy contamination and cascade-driven spatial gradients. These findings support the development of evidence-based, species-specific fish-consumption advisories that account for trophic position, body size, and vulnerable population groups in cascade reservoir systems affected by legacy contamination.

## Figures and Tables

**Table 1:** Overview of sampled species, trophic guild, reservoirs, and median morphometrics.

Species	Common Name	Trophic Guild	Reservoir Distribution (K, SK, I)	*n*	TL Median (cm)	TW Median (g)
*Cyprinus carpio*	Common carp	Omnivore	K = 0, SK = 3, I = 0	3	48.1	2416
*Squalius orpheus*	Orpheus dace	Omnivore	K = 0, SK = 0, I = 3	3	34.6	609
*Vimba melanops*	Macedonian vimba	Omnivore	K = 5, SK = 0, I = 0	5	23.6	181
*Silurus glanis*	European catfish	Predator	K = 0, SK = 3, I = 0	3	62.0	1862
*Perca fluviatilis*	European perch	Predator	K = 5, SK = 0, I = 4	9	24.2	235
*Carassius gibelio*	Prussian carp	Omnivore	K = 5, SK = 4, I = 8	17	28.4	451
*Rutilus rutilus*	Roach	Omnivore	K = 5, SK = 0, I = 4	9	22.7	196

Note: Detailed per-reservoir morphometric statistics (medians and IQRs) are provided in Table S1. Abbreviations: K—Kardzhali; SK—Studen Kladenets; I—Ivaylovgrad.

**Table 2:** Tissue-specific THg (mg·kg^−1^ ww): mean ± SD, median, range and sample size (*n*).

Organ	Mean ± SD (mg·kg^−1^)	Median	Min	Max	*n*
Bones	0.023 ± 0.018	0.016	0.002	0.069	49
Gills	0.011 ± 0.011	0.008	0.001	0.042	49
Gonads	0.006 ± 0.005	0.005	0.001	0.017	49
Hearts	0.024 ± 0.031	0.011	0.001	0.124	49
Kidneys	0.020 ± 0.021	0.011	0.003	0.107	49
Liver	0.032 ± 0.051	0.010	0.002	0.198	49
Muscle	0.044 ± 0.034	0.032	0.008	0.116	49
Skin	0.016 ± 0.012	0.015	0.001	0.067	49
Spleen	0.022 ± 0.027	0.007	0.000	0.120	49

**Table 3:** Pearson correlations between morphometric predictors (TL, TW) and tissue THg across organs and species.

Organ	Pearson r (TL vs. THg)	Pearson r (TW vs. THg)	*n*
Bones	−0.302	−0.326	49
Gills	−0.284	−0.306	49
Gonads	0.245	0.087	49
Hearts	−0.220	−0.236	49
Kidneys	−0.159	−0.164	49
Liver	−0.155	−0.157	49
Muscle	−0.172	−0.230	49
Skin	−0.334	−0.364	49
Spleen	−0.203	−0.230	49

**Table 4:** (**A**) Deterministic THQ (IR = 140 g week^−1^) and SWI (median and P95) by species. (**B**) Monte Carlo THQ (*N* = 30,000): P50, P95, and P(THQ > 1) by species and consumer group (IR~Tri(50, 140, 350) g·week^−1^).

**(A)**									
**Species**	**THQ_det (Adults)**	**THQ_det (Pregnant)**	**THQ_det (Children)**	**SWI_med (Adults)**	**SWI_med (Pregnant)**	**SWI_med (Children)**	**SWI_P95 (Adults)**	**SWI_P95 (Pregnant)**	**SWI_P95 (Children)**
1	0.306	0.357	1.428	850	728	182	788	676	169
2	0.125	0.146	0.583	2081	1784	446	1797	1540	385
3	0.119	0.139	0.557	2180	1869	467	1571	1346	337
4	0.082	0.096	0.383	3165	2713	678	2243	1922	481
5	0.051	0.059	0.236	5132	4399	1100	1380	1183	296
6	0.066	0.077	0.308	3940	3377	844	2162	1853	463
7	0.063	0.073	0.294	4130	3540	885	3014	2583	646
8	0.025	0.029	0.116	10,430	8940	2235	7637	6546	1636
**(B)**									
**Species**	**MC P50 (Adults)**	**MC P50 (Pregnant)**	**MC P50 (Children)**	**MC P95 (Adults)**	**MC P95 (Pregnant)**	**MC P95 (Children)**	**MC P(>1) % (Adults)**	**MC P(>1) % (Pregnant)**	**MC P(>1) % (Children)**
1	0.033	0.038	0.153	0.063	0.073	0.294	0.0%	0.0%	0.0%
2	0.079	0.092	0.367	0.159	0.186	0.743	0.0%	0.0%	0.1%
3	0.140	0.164	0.656	0.289	0.335	1.346	0.0%	0.0%	18.7%
4	0.145	0.171	0.682	0.276	0.322	1.277	0.0%	0.0%	17.6%
5	0.369	0.432	1.740	0.642	0.756	3.021	0.0%	0.0%	90.1%
6	0.080	0.093	0.374	0.274	0.319	1.303	0.0%	0.0%	11.5%
7	0.086	0.100	0.402	0.199	0.232	0.935	0.0%	0.0%	3.6%

1—Perch, 2—European catfish, 3—Macedonian vimba, 4—Prussian carp large, 5—Prussian carp small, 6—Roach, 7—Orpheus dace, 8—Carp. Notes: THQ was calculated using IR = 140 g week^−1^ (0.02 kg day^−1^), RfD = 0.1 µg kg^−1^ day^−1^, and BW = 70/60/15 kg (Adults/Pregnant/Children). SWI was calculated using the EFSA TWI of 1.3 µg kg^−1^ week^−1^. 1—Carp, 2—Orpheus dace, 3—Macedonian vimba, 4—European catfish, 5—Perch, 6—Prussian carp, 7—Roach. Notes: Monte Carlo settings: *N* = 30,000; C sampled via species-specific empirical bootstrap of muscle THg (mg·kg^−1^ ww); IR~Tri(50, 140, 350) g week^−1^ (converted to daily for THQ); BW = 70/60/15 kg (Adults/Pregnant/Children); RfD (MeHg) = 0.0001 mg kg^−1^ day^−1^ (=0.1 µg kg^−1^ day^−1^). Species taxonomy: “Prussian carp” includes records labelled as “Prussian carp (large)”.

## Data Availability

The original contributions presented in this study are included in the article/Supplementary Materials. Further inquiries can be directed to the corresponding author.

## Supplementary Materials

The following supporting information can be downloaded at: https://www.mdpi.com/article/10.3390/toxics14040291/s1. Table S1: Species × Reservoir TL/TW (medians, IQR). Table S2: Summary statistics (Species × Organ) for THg (mg kg^−1^ ww). Table S3: Organ-to-organ Pearson correlation matrix for tissue THg (mg kg^−1^ ww). Table S4: Reservoir-specific Pearson correlations for TL/TW vs. muscle THg. Table S5: One-way ANOVA for muscle THg across reservoirs. Table S6: Monte Carlo THQ percentiles and exceedance probabilities by species and consumer group. Table S7: Hazard Index (HI)—deterministic results (ΣTHQ per basket) by consumer group. Figure S1: Safe Weekly Intake (SWI) for children (15 kg), based on muscle THg: (a) SWI calculated from median muscle THg (mg kg^−1^ ww); (b) SWI calculated from P95 muscle THg (mg kg^−1^ ww)—conservative estimate. Color scale: Green ≥ 300 g week^−1^; Yellow 140–299 g week^−1^; Red < 140 g week^−1^. Figure S2: Monte Carlo THQ suites (PDF distributions) for all studied species across three consumer groups (Adults 70 kg; Pregnant women 60 kg; Children 15 kg). Dashed line indicates THQ = 1. IR ~ Tri(50, 140, 350) g week^−1^; RfD = 0.1 µg kg^−1^ day^−1^. Figure S3: Hazard Index (HI = ΣTHQ) distributions for diet baskets across consumer groups: (a) HI density distributions (PDF); (b) HI cumulative distributions (CDF). Figure S4: Tissue–species clustermaps (row-normalized THg): (a) Tissue × Species clustermap; (b) Species × Tissue clustermap. Color bars indicate trophic guilds: predator (red), omnivore (blue). Figure S5: Tissue × Reservoir patterns of THg across the Arda cascade: (a) Row-normalized clustermap (z-score), showing hierarchical grouping of tissues and reservoirs; (b) Median THg (mg kg^−1^ ww) heatmap for each tissue × reservoir combination. Figure S6: Size–Hg relationships across reservoirs (species-specific panels): (a) TW vs. muscle THg by reservoir, with OLS fit and 95% CI; each panel reports r, *p*, and n; (b) TL vs. muscle THg by reservoir, with OLS fit and 95% CI; each panel reports r, *p*, and n. Figure S7: Length–mercury (TL–THg) relationships across reservoirs: (a) Prussian carp TL–muscle THg for Kardzhali, Studen Kladenets, and Ivaylovgrad, with OLS fit and 95% CI; (b) Split scatterplots for perch, Prussian carp, and roach across the three reservoirs, each panel showing OLS fit, 95% CI, r, *p*, and n. Figure S8: Monte Carlo-based Target Hazard Quotient (THQ) for fish species across three consumer groups (Adults 70 kg; Pregnant women 60 kg; Children 15 kg). Points represent median (P50) THQ, and horizontal whiskers denote upper bound (P95). The dashed red line marks the safety threshold (THQ = 1).

## Abbreviations

The following abbreviations are used in this manuscript:

ANOVA	Analysis of Variance
BW	Body Weight (kg)
CDF	Cumulative Distribution Function
CI	Confidence Interval
DOC	Dissolved Organic Carbon
EFSA	European Food Safety Authority
Hg	Mercury
HI	Hazard Index (HI = ΣTHQi)
LOD	Limit of Detection
MC	Monte Carlo (probabilistic simulation)
MeHg	Methylmercury
n	Sample size
P50	Median (50th percentile)
P95	95th percentile
PDF	Probability Density Function
PCA	Principal Component Analysis
RfD	Reference Dose (0.1 μg·kg^−1^·day^−1^ for MeHg)
SD	Standard Deviation
SWI	Safe Weekly Intake (g·week^−1^)
THg	Total Mercury
THQ	Target Hazard Quotient
TL	Total Length (cm)
TWI	Tolerable Weekly Intake (EFSA = 1.3 μg·kg^−1^·week^−1^)
TW	Total Weight (g)
WW	Wet Weight
ΣTHQi	Sum of individual tissue-specific THQ values
