# Supplementary material for: Tissue-Specific Mercury Bioaccumulation and Probabilistic Human Health Risk in Freshwater Fish from the Arda River Reservoir Cascade (Bulgaria)
Source: Toxics. 2026 Mar 28;14(4):291. doi: 10.3390/toxics14040291 (PMC13119662; doi:10.3390/toxics14040291)
Supplement: Supplementary file 1 [file toxics-14-00291-s001.zip › toxics-4196778-supplementary.pdf]

Supplementary Tables

Table S1. Species composition, reservoir distribution (n), and fish size by reservoir (median, IQR). Medians and interquartile ranges (IQRs) for total length (TL, cm) and total weight (TW, g) are reported per species and reservoir.

Species	*n*	Reservoirs (K, SK, I)	TL median (IQR), cm	TW median (IQR), g
Common carp	3	K=0, SK=3, I=0	48.10 (48.05–49.35)	2416.0 (2358.0–2419.0)
European catfish	3	K=0, SK=3, I=0	62.00 (61.75–70.00)	1862.0 (1742.0–2585.0)
European perch	5	K=5, SK=0, I=0	24.00 (23.70–24.20)	240.90 (231.89–254.92)
European perch	4	K=0, SK=0, I=4	24.60 (24.20–25.15)	232.81 (223.89–256.12)
Macedonian vimba	5	K=5, SK=0, I=0	23.60 (23.50–25.80)	180.94 (156.50–221.44)
Orpheus dace	3	K=0, SK=0, I=3	34.60 (34.00–36.05)	608.65 (549.60–619.45)
Prussian carp	9	K=5, SK=4, I=0	29.20 (28.40–31.60)	466.13 (446.04–506.18)
Prussian carp (large)	4	K=0, SK=0, I=4	29.80 (26.83–31.60)	513.21 (410.44–576.61)
Prussian carp (small)	4	K=0, SK=0, I=4	20.30 (19.98–21.70)	147.80 (143.72–152.22)
Roach	9	K=5, SK=0, I=4	22.70 (22.60–24.20)	195.57 (178.84–211.01)

Note: K — Kardzhali; SK — Studen Kladenets; I — Ivaylovgrad.

Table S2. Summary statistics (Species × Organ) for THg (mg.kg^{-1}, ww).

**Species**	**Organ**	***n***	**mean**	**Sd**	**median**	**min**	**max**
Carp	Bones	3	0.005	0.003	0.003	0.002	0.008
Carp	Gills	3	0.002	0.000	0.002	0.001	0.002
Carp	Gonads	3	0.001	0.002	0.000	0.000	0.003
Carp	Hearts	3	0.003	0.004	0.001	0.001	0.007
Carp	Kidneys	3	0.009	0.005	0.009	0.004	0.014
Carp	Liver	3	0.005	0.003	0.005	0.002	0.008
Carp	Muscle	3	0.010	0.002	0.009	0.008	0.012
Carp	Skin	3	0.003	0.001	0.002	0.001	0.004
Carp	Spleen	3	0.003	0.000	0.003	0.002	0.003
Orpheus dace	Bones	3	0.009	0.006	0.007	0.005	0.016
Orpheus dace	Gills	3	0.008	0.004	0.009	0.004	0.012
Orpheus dace	Gonads	3	0.005	0.007	0.001	0.001	0.013
Orpheus dace	Hearts	3	0.005	0.004	0.007	0.001	0.008
Orpheus dace	Kidneys	3	0.011	0.004	0.011	0.007	0.015
Orpheus dace	Liver	3	0.007	0.004	0.007	0.004	0.012
Orpheus dace	Muscle	3	0.023	0.007	0.022	0.017	0.031
Orpheus dace	Skin	3	0.011	0.002	0.010	0.009	0.013
Orpheus dace	Spleen	3	0.012	0.014	0.004	0.004	0.029

Macedonian vimba	Bones	5	0.021	0.007	0.017	0.015	0.029
Macedonian vimba	Gills	5	0.016	0.004	0.014	0.013	0.022
Macedonian vimba	Gonads	5	0.007	0.001	0.008	0.005	0.009
Macedonian vimba	Hearts	5	0.030	0.006	0.031	0.022	0.036
Macedonian vimba	Kidneys	5	0.020	0.009	0.015	0.013	0.030
Macedonian vimba	Liver	5	0.035	0.022	0.031	0.015	0.072
Macedonian vimba	Muscle	5	0.042	0.013	0.042	0.025	0.061
Macedonian vimba	Skin	5	0.020	0.004	0.019	0.015	0.024
Macedonian vimba	Spleen	5	0.052	0.025	0.045	0.028	0.095
European catfish	Bones	3	0.015	0.002	0.016	0.013	0.017
European catfish	Gills	3	0.007	0.003	0.006	0.005	0.011
European catfish	Gonads	3	0.015	0.002	0.015	0.013	0.017
European catfish	Hearts	3	0.019	0.013	0.017	0.008	0.033
European catfish	Kidneys	3	0.017	0.003	0.018	0.014	0.019
European catfish	Liver	3	0.029	0.006	0.030	0.023	0.034
European catfish	Muscle	3	0.042	0.010	0.044	0.031	0.051
European catfish	Skin	3	0.009	0.003	0.009	0.006	0.012
European catfish	Spleen	3	0.019	0.002	0.020	0.017	0.021
Perch	Bones	9	0.056	0.013	0.063	0.040	0.069
Perch	Gills	9	0.029	0.010	0.031	0.014	0.042
Perch	Gonads	9	0.010	0.003	0.011	0.006	0.016
Perch	Hearts	9	0.081	0.029	0.097	0.044	0.124
Perch	Kidneys	9	0.056	0.027	0.060	0.023	0.107
Perch	Liver	9	0.116	0.071	0.131	0.019	0.198
Perch	Muscle	9	0.106	0.010	0.107	0.088	0.116
Perch	Skin	9	0.031	0.016	0.027	0.014	0.067
Perch	Spleen	9	0.058	0.032	0.063	0.028	0.120
Prussian carp	Bones	17	0.014	0.007	0.011	0.006	0.033
Prussian carp	Gills	17	0.003	0.001	0.002	0.002	0.007
Prussian carp	Gonads	17	0.002	0.001	0.002	0.001	0.004

Prussian carp	Hearts	17	0.007	0.004	0.007	0.001	0.018
Prussian carp	Kidneys	17	0.008	0.003	0.008	0.003	0.014
Prussian carp	Liver	17	0.006	0.003	0.006	0.002	0.010
Prussian carp	Muscle	17	0.030	0.020	0.018	0.011	0.085
Prussian carp	Skin	17	0.012	0.006	0.010	0.003	0.022
Prussian carp	Spleen	17	0.005	0.002	0.004	0.000	0.009
Roach	Bones	9	0.021	0.009	0.019	0.006	0.034
Roach	Gills	9	0.012	0.005	0.012	0.001	0.018
Roach	Gonads	9	0.007	0.004	0.005	0.002	0.014
Roach	Hearts	9	0.013	0.005	0.013	0.004	0.022
Roach	Kidneys	9	0.012	0.005	0.011	0.004	0.019
Roach	Liver	9	0.013	0.005	0.011	0.005	0.020
Roach	Muscle	9	0.026	0.010	0.023	0.014	0.045
Roach	Skin	9	0.016	0.008	0.017	0.003	0.030
Roach	Spleen	9	0.014	0.010	0.021	0.003	0.024

Table S3. Organ-to-organ Pearson correlation matrix for tissue THg (mg.kg^{-1}, ww).

Organ	Bones	Gills	Gonads	Hearts	Kidneys	Liver	Muscle	Skin	Spleen
Bones	1.0	0.856	0.518	0.842	0.806	0.751	0.84	0.711	0.679
Gills	0.856	1.0	0.525	0.912	0.849	0.892	0.799	0.563	0.844
Gonads	0.518	0.525	1.0	0.479	0.436	0.389	0.517	0.399	0.467
Hearts	0.842	0.912	0.479	1.0	0.924	0.954	0.861	0.563	0.85
Kidneys	0.806	0.849	0.436	0.924	1.0	0.902	0.777	0.494	0.8
Liver	0.751	0.892	0.389	0.954	0.902	1.0	0.762	0.412	0.825
Muscle	0.84	0.799	0.517	0.861	0.777	0.762	1.0	0.625	0.672
Skin	0.711	0.563	0.399	0.563	0.494	0.412	0.625	1.0	0.483
Spleen	0.679	0.844	0.467	0.85	0.8	0.825	0.672	0.483	1.0

Table S4. Reservoir-specific Pearson correlations for TL/TW vs muscle THg.

Reservoir	r(TL, muscle Hg)	*n*(TL)	r(TW, muscle Hg)	*n*(TW)
Ivaylovgrad	-0.104	19	-0.165	19
Kardzhali	0.090	20	0.146	20
Studen Kladenets	0.556	10	0.116	10

Table S5. One-way ANOVA for muscle THg across reservoirs.

**Reservoir**	***n***	**mean**	**sd**
Ivaylovgrad	19	0.0393	0.035
Kardzhali	20	0.0581	0.0336
Studen Kladenets	10	0.0225	0.0149

Table S6. Monte Carlo THQ percentiles and exceedance probability by species and group.

Group	Species	P50_THQ	P95_THQ	P(THQ>1)
Adults	Carp	0.03	0.06	0.0%
Adults	Orpheus dace	0.08	0.16	0.0%
Adults	Macedonian vimba	0.14	0.29	0.0%
Adults	European catfish	0.15	0.27	0.0%
Adults	Perch	0.37	0.65	0.0%
Adults	Prussian carp	0.08	0.28	0.0%
Adults	Roach	0.09	0.20	0.0%
Children	Carp	0.15	0.30	0.0%
Children	Orpheus dace	0.37	0.75	0.1%
Children	Macedonian vimba	0.66	1.34	18.9%
Children	European catfish	0.68	1.29	17.8%
Children	Perch	1.73	3.01	90.3%
Children	Prussian carp	0.37	1.31	11.7%
Children	Roach	0.40	0.94	3.6%
Pregnant	Carp	0.04	0.07	0.0%
Pregnant	Orpheus dace	0.09	0.19	0.0%
Pregnant	Macedonian vimba	0.16	0.34	0.0%
Pregnant	European catfish	0.17	0.32	0.0%
Pregnant	Perch	0.43	0.75	0.0%
Pregnant	Prussian carp	0.09	0.32	0.0%
Pregnant	Roach	0.10	0.23	0.0%

Note: Monte Carlo (30,000 iterations); C from species-specific empirical bootstrap of muscle THg (mg·kg^{-1} ww); IR - Tri(50, 140, 350) g·week^{-1} (converted to daily for THQ); BW = 70/60/15 kg (Adults/Pregnant/Children); RfD = 0.1 µg kg^{-1} day^{-1} (= 0.0001 mg kg^{-1} day^{-1}).

Results are reported as P50, P95, and P(THQ > 1). “Prussian carp” includes records labeled “Prussian carp (large)”.

Table S7. Hazard Index (HI) — Deterministic results (ΣTHQ per basket) by consumer group.

Basket	Adults	Pregnant	Children
Catfish_140	0.121	0.141	0.562
Custom_Perch100x2_Roach150	0.513	0.598	2.392
LowRisk_140	0.088	0.103	0.411
Mixed_70_70	0.195	0.228	0.911
Predator_140	0.302	0.353	1.410

Note: HI_det computed with IR anchored to 140 g/week for weekly baskets as specified; values ≥ 1 indicate potential non-carcinogenic concern.

Supplementary Figures

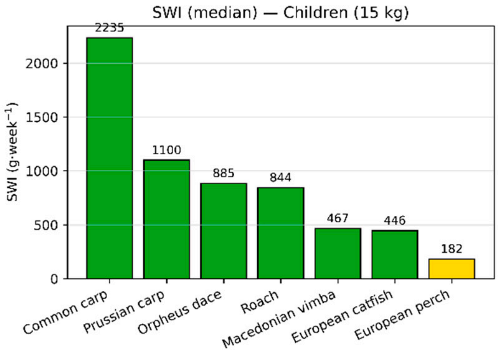

(**a**) SWI calculated from median muscle THg (mg.kg^{-1} ww)

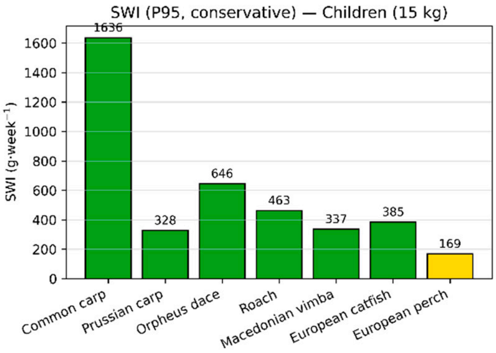

(**b**) SWI calculated from P95 muscle THg (mg.kg^{-1} ww)

Figure S1. Safe Weekly Intake (SWI) — Children (15 kg), based on muscle THg: (**a**) SWI calculated from median muscle THg (mg.kg^{-1} ww); (**b**) SWI calculated from P95 muscle THg (mg.kg^{-1} ww); conservative estimate. Color scale: Green ≥ 300 g.week^{-1}, Yellow 140–299 g.week^{-1}, Red < 140 g.week^{-1}.

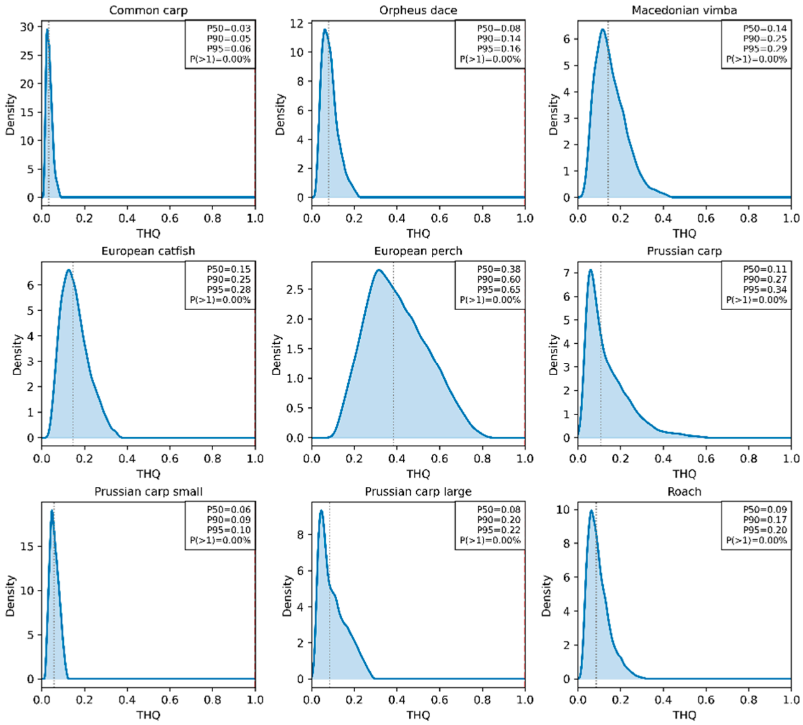

(a)Adults (70 kg)

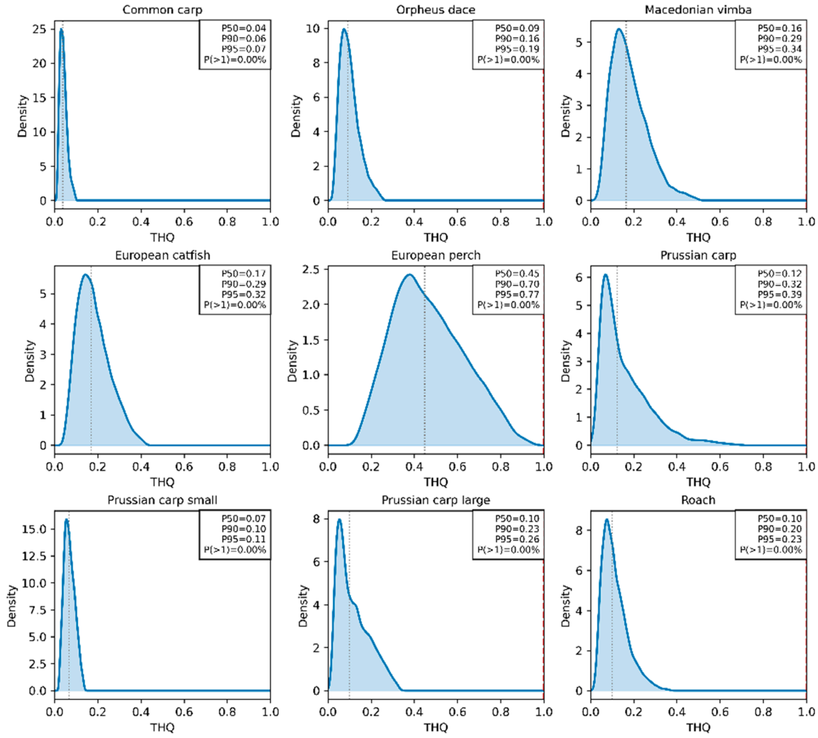

(b)Pregnant women (60 kg)

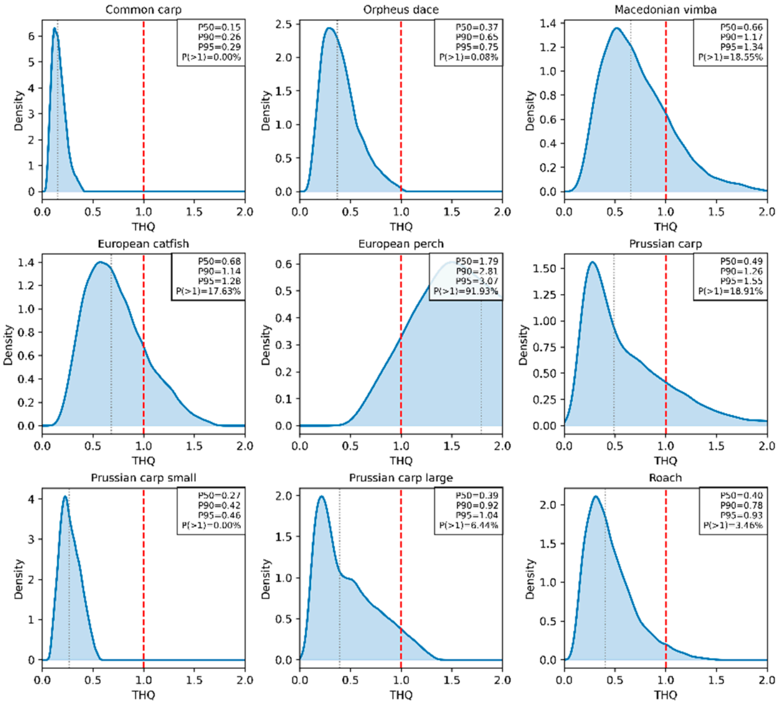

c) Children (15 kg)

Figure S2. Monte Carlo THQ suites (PDF distributions) for all studied species across three consumer groups (a)Adults (70 kg); (b)Pregnant women (60 kg); (c) Children (15 kg). Dashed line indicates THQ = 1. IR ~ Tri(50,140,350) g.week^{-1}; RfD = 0.1 μg.kg^{-1}day^{-1}.

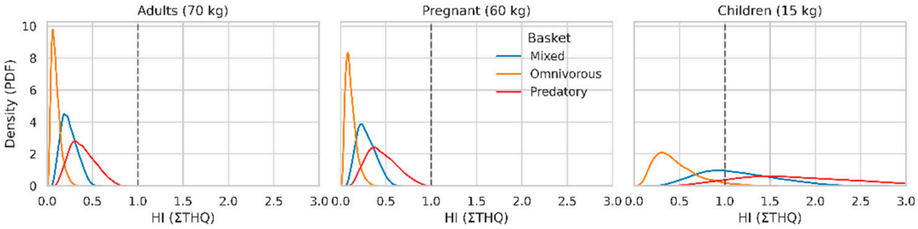

(a) HI density distributions (PDF)

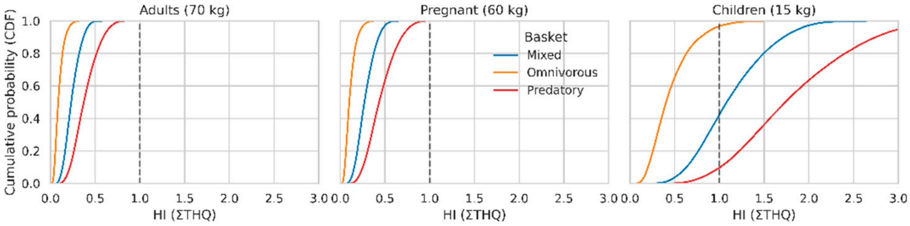

(**b**) HI cumulative distributions (CDF)

Figure S3. Hazard Index (HI = ΣTHQ) distributions for diet baskets across consumer groups: (**a**) HI density distributions (PDF); (**b**) HI cumulative distributions (CDF).

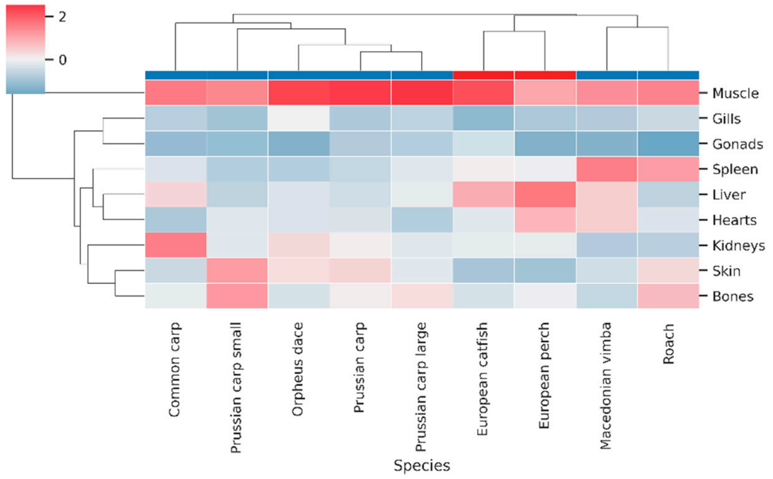

(a) Tissue × Species clustermap

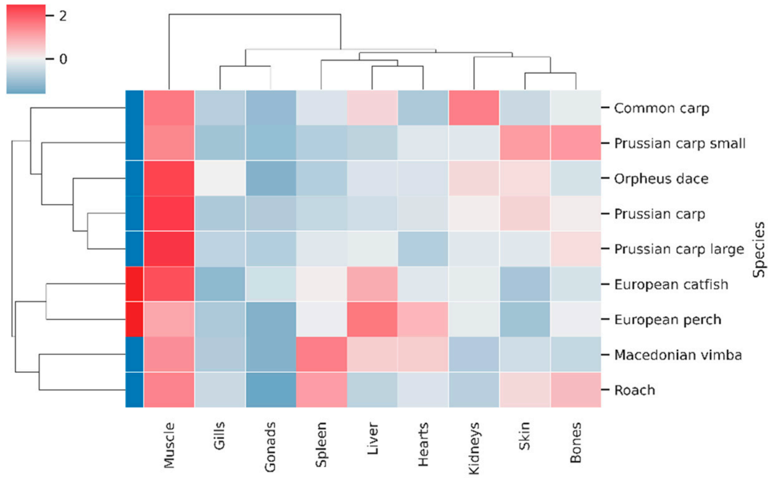

(b) Species × Tissue clustermap

Figure S4. Tissue–species clustermaps (row-normalized THg): (**a**) Tissue × Species clustermap; (**b**) Species × Tissue clusterma, Color bars indicate trophic guild: predator (red), omnivore (blue).

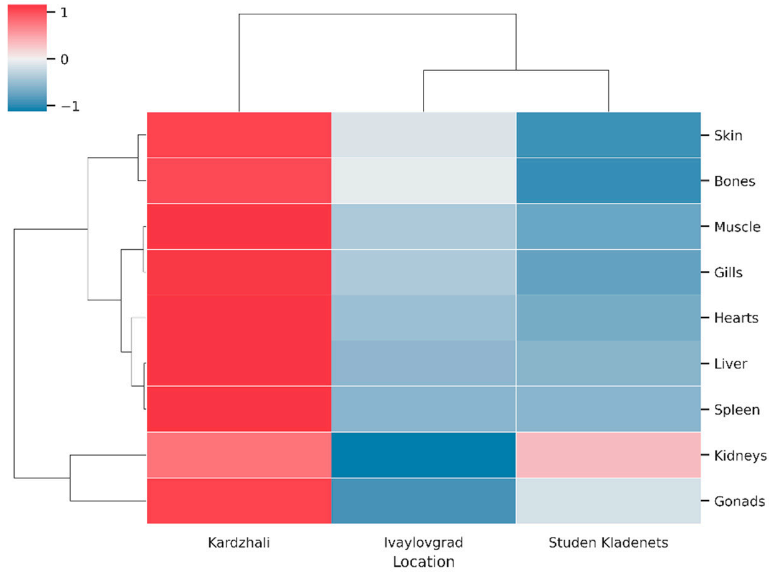

(a) Row-normalized clustermap (z-score), showing hierarchical grouping of tissues and reservoirs.

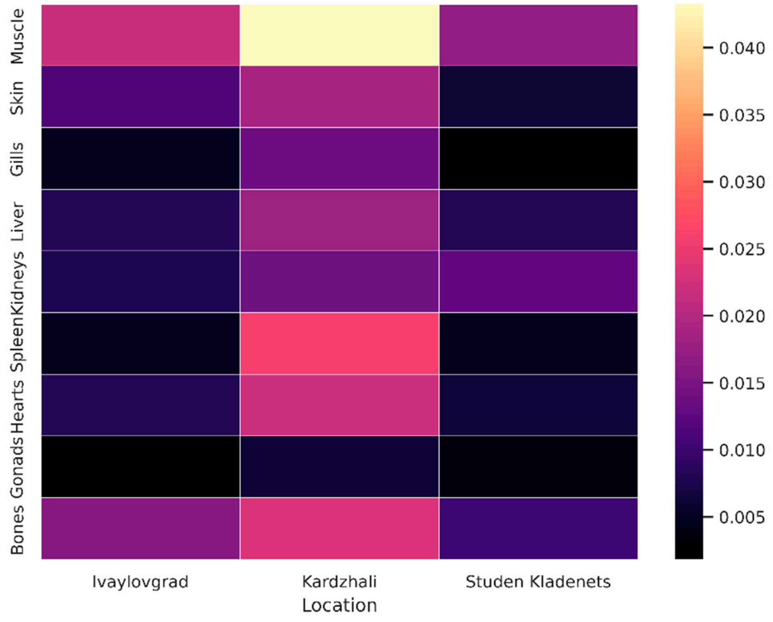

(b) Median THg (mg.kg-1 ww) heatmap for each tissue × reservoir combination

Figure S5. Tissue × Reservoir patterns of THg across the Arda cascade: (**a**) Row-normalized clustermap (z-score), showing hierarchical grouping of tissues and reservoirs.; (**b**) Median THg (mg.kg^{-1} ww) heatmap for each tissue × reservoir combination.

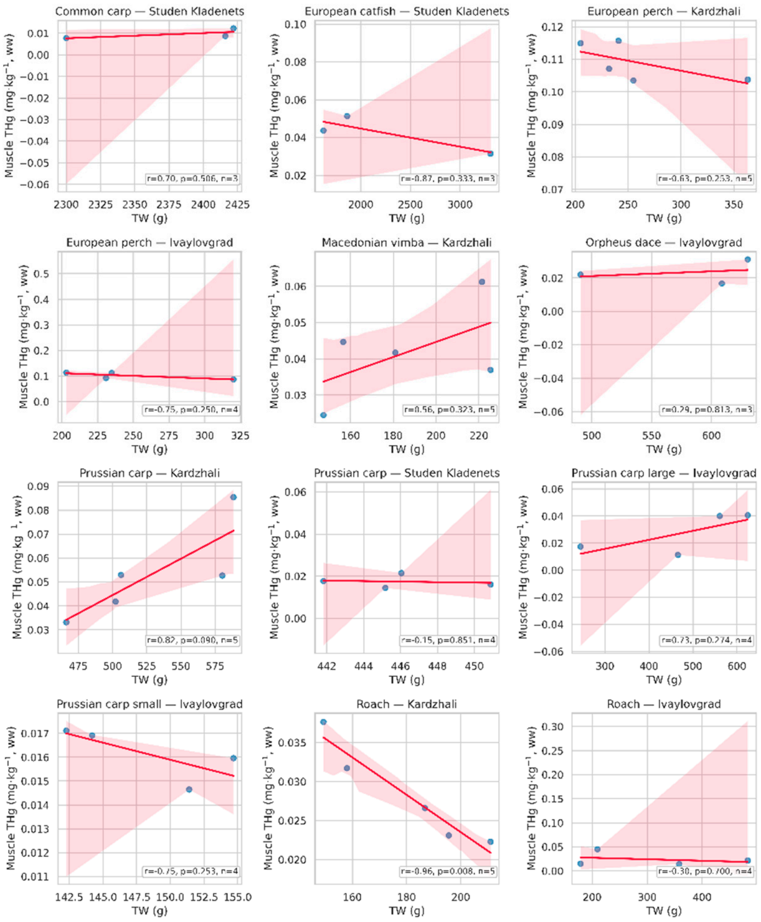

a) TW vs muscle THg by reservoir, with OLS fit and 95% CI; each panel reports r, p, and n.

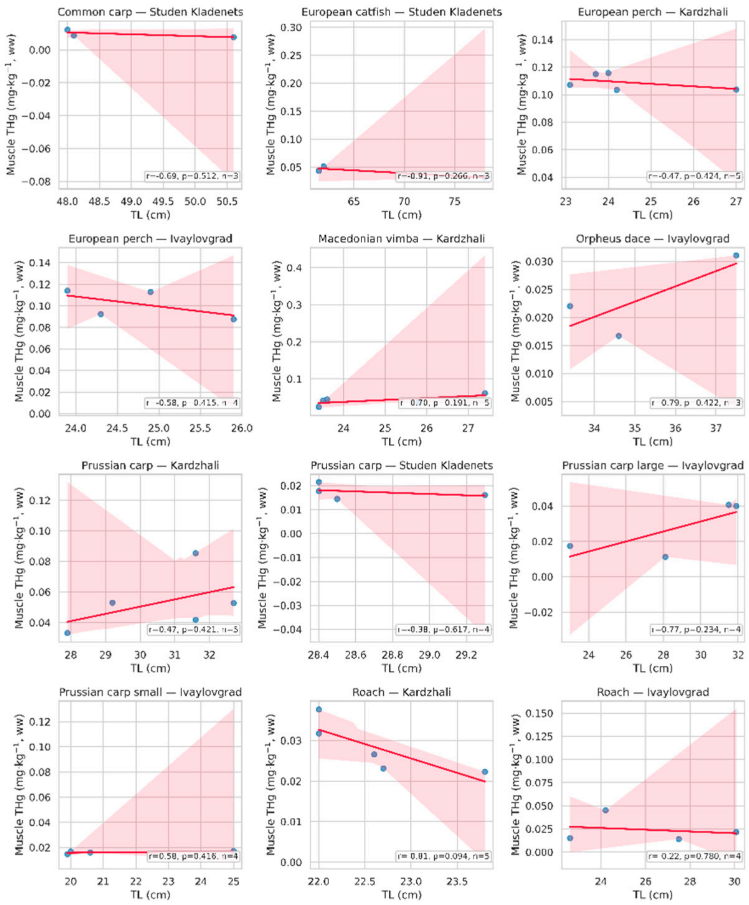

(b) TL vs muscle THg by reservoir, with OLS fit and 95% CI; each panel reports r, p, and n Total length (TL) vs THg

Figure S6. Size–Hg relationships across reservoirs (species-specific panels)**:** (**a**) TW vs muscle THg by reservoir, with OLS fit and 95% CI; each panel reports *r*, *p*, and *n*.; (**b**) TL vs muscle THg by reservoir, with OLS fit and 95% CI; each panel reports *r*, *p*, and *n* Total length (TL) vs THg (Full matrix diagnostic)

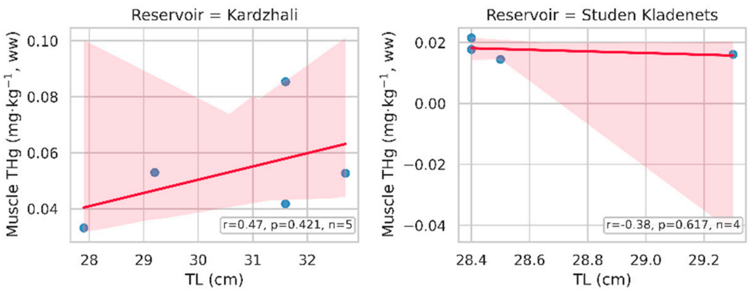

(**a**) Prussian carp TL–muscle THg for Kardzhali, Studen Kladenets, and Ivaylovgrad, with OLS fit and 95% CI

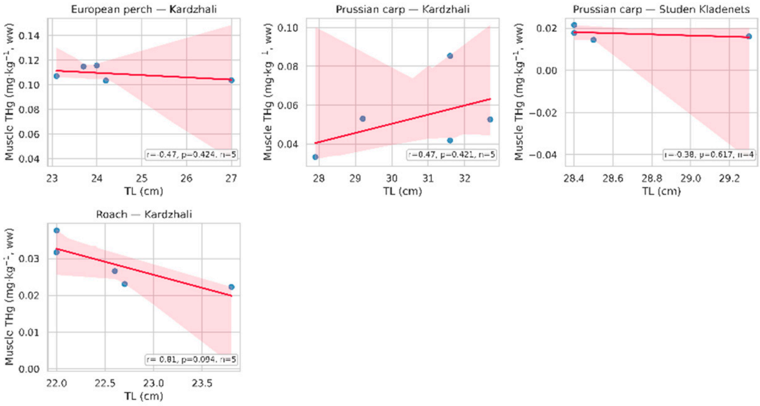

(b) Split scatterplots for Perch, Prussian carp, and Roach across the three reservoirs, each panel showing OLS fit, 95% CI, r, p, and n

Figure S7. Length–mercury (TL–THg) relationships across reservoirs: (**a**) Prussian carp TL–muscle THg for Kardzhali, Studen Kladenets, and Ivaylovgrad, with OLS fit and 95% CI.; (**b**) Split scatterplots for *Perch*, *Prussian carp*, and *Roach* across the three reservoirs, each panel showing OLS fit, 95% CI, *r*, *p*, and *n*.

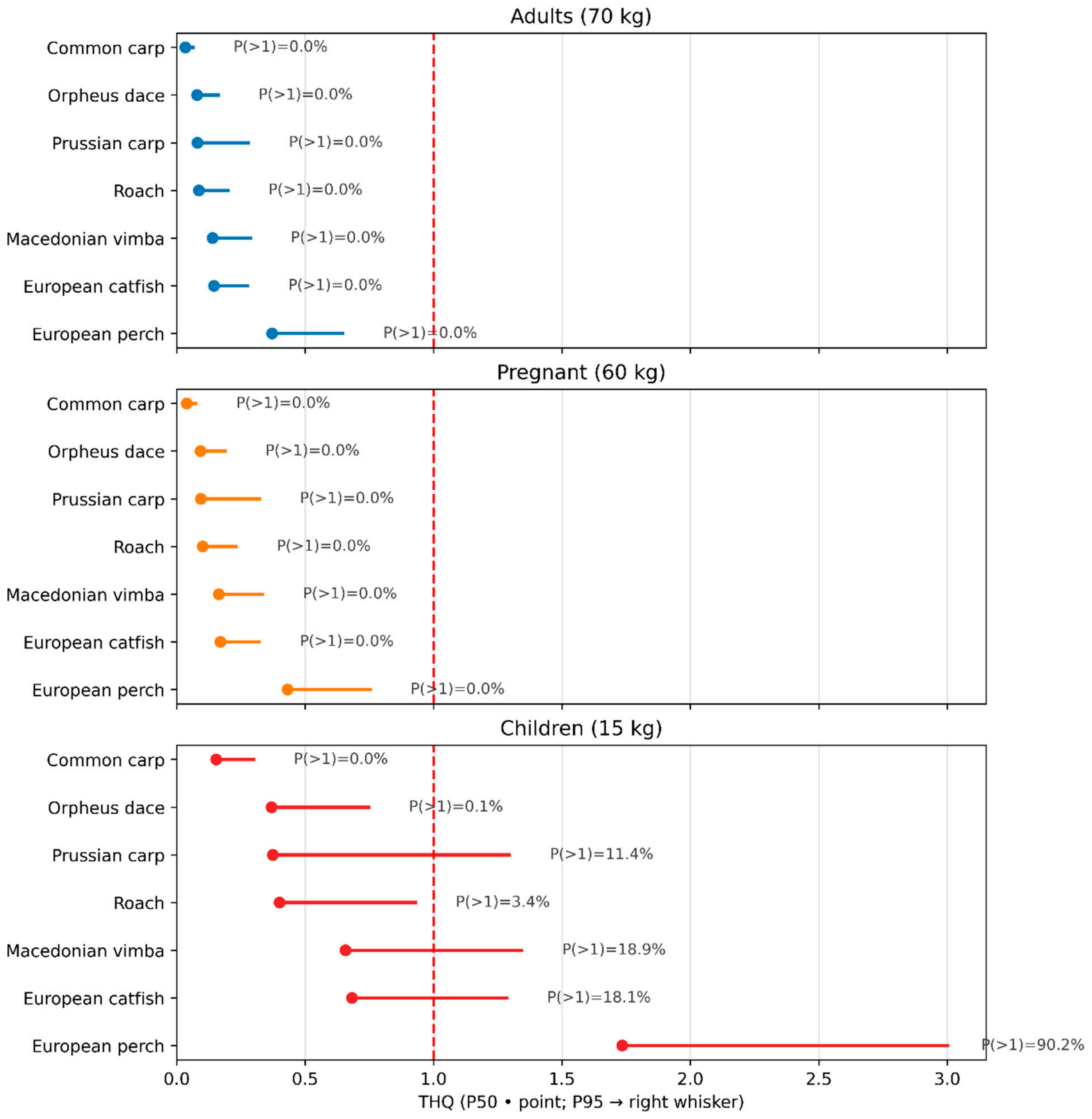

Figure S8. Monte Carlo–based Target Hazard Quotient (THQ) for different fish species across three consumer groups (Adults 70 kg, Pregnant women 60 kg, Children 15 kg). Points represent the median (P50) THQ, and horizontal whiskers denote the upper bound (P95). The dashed red line marks the safety threshold (THQ = 1).
